# The epidemiology of symptomatic midfoot osteoarthritis in community-dwelling older adults: cross-sectional findings from the Clinical Assessment Study of the Foot

**DOI:** 10.1186/s13075-015-0693-3

**Published:** 2015-07-13

**Authors:** Martin J. Thomas, George Peat, Trishna Rathod, Michelle Marshall, Andrew Moore, Hylton B. Menz, Edward Roddy

**Affiliations:** Arthritis Research UK Primary Care Centre, Research Institute for Primary Care and Health Sciences, Keele University, Keele, Staffordshire ST5 5BG UK; Musculoskeletal Research Unit, School of Clinical Sciences, University of Bristol, Bristol, BS10 5NB UK; Lower Extremity and Gait Studies Program, School of Allied Health, La Trobe University, Bundoora, Victoria 3086 Australia

## Abstract

**Introduction:**

The foot is largely overlooked in calls for better characterisation of clinical phenotypes in osteoarthritis (OA). Yet the midfoot complex in particular has the potential to provide important insights into OA pathogenesis given its central role in lower limb load transmission and alignment. Its recent inclusion in radiographic atlases has paved the way for international studies. In this UK study, we provide the first comprehensive account of the descriptive epidemiology of symptomatic midfoot OA.

**Methods:**

Participants aged ≥50 years registered with four general practices were recruited via a mailed health survey (n = 5109 responders) and research clinic (n = 560 responders). Symptomatic midfoot OA was defined as midfoot pain in the last 4 weeks, combined with radiographic OA in one or more joints (1st and 2nd cuneometatarsal, navicular first cuneiform and talonavicular joints) graded from weight-bearing dorso-plantar and lateral radiographs using a validated atlas. Prevalence estimates, overall and stratified by age, gender, and socio-economic class, were derived using multiple imputation and weighted logistic regression. Associations between symptomatic midfoot OA and current body mass index, previous injury, history of high-heeled footwear, nodal interphalangeal joint OA and patterns of comorbidity were estimated using binary logistic regression. Healthcare use was summarised.

**Results:**

Symptomatic midfoot OA was present in 12.0 % (95 % CI: 10.9, 13.2) of the population aged over 50 years. Higher occurrence was observed in females, adults aged over 75 years, and those in intermediate/routine occupational classes. Obesity, previous foot/ankle injury, and pain in other weight-loaded joints, but not high-heeled footwear or nodal interphalangeal joint OA, were associated with increased risk of symptomatic midfoot OA. Persons with symptomatic midfoot OA were also more likely to report multiple non-musculoskeletal comorbidities, including diabetes. In the previous 12 months, the proportions consulting a general practitioner, physiotherapist or podiatrist/chiropodist about foot pain were 46.2 %, 18.5 % and 47.9 % respectively. A total of 64.7 % had used oral analgesia in the past month for foot pain (36.1 % paracetamol, 31.9 % mild/moderate opioids, 27.7 % NSAIDs).

**Conclusions:**

Our study confirms that symptomatic OA frequently affects the midfoot. The patterns of associations are interpreted as being largely consistent with the role of mechanical factors in its pathogenesis.

**Electronic supplementary material:**

The online version of this article (doi:10.1186/s13075-015-0693-3) contains supplementary material, which is available to authorized users.

## Introduction

Symptomatic osteoarthritis (OA) is now widely accepted as being a multifactorial pathology affecting the whole joint complex [1]. The different aetiology, risk factors and prognosis seen across different joint sites, such as the hip, knee and hand, suggest that characterisation of discrete clinically relevant OA phenotypes is necessary to enable targeted treatment interventions to be developed [2].

Although the foot has largely been neglected relative to other sites commonly affected by OA [3], persistent foot pain is common in older adults [4], with approximately one in six estimated to have symptomatic foot OA [5]. As with other regions of the body, OA of the foot joint complex may comprise more than one phenotype. Recent latent class analysis of radiographic foot OA at five key sites (1st metatarsophalangeal joint (MTPJ), 1st and 2nd cuneometatarsal joint (CMJs), navicular first cuneiform joint (NCJ) and talonavicular joint (TNJ)) identified an isolated bilateral 1st MTPJ OA group and a polyarticular midfoot-dominant OA group as possible distinct radiographic phenotypes [6]. In the foot, the predominant focus of epidemiological studies has been on OA at the 1st MTPJ [3]. However, better characterisation of a symptomatic midfoot OA phenotype could facilitate more targeted clinical assessment and management, particularly as primary care diagnosis of foot symptoms is often limited and unclear [7, 8].

The midfoot also presents a particularly interesting complex in which to investigate OA given its important load distribution function [9], enabling the foot to conform to terrain and yet provide a rigid lever for forward motion when walking. It remains highly speculative whether OA in the midfoot has important implications for the development of OA in adjacent joints. Case series reporting high rates of OA in adjacent joints following midfoot fusion (for example, [10, 11]) have lacked appropriate control observations and may be confounded by factors associated with both midfoot fusion and OA in other foot joints.

The objectives of this study were to: (i) provide population prevalence estimates for symptomatic midfoot OA (including estimates for midfoot pain and disabling symptomatic midfoot OA) in adults aged 50 years and over, (ii) examine the association with selected potential aetiological factors, (iii) describe associated patterns of comorbidity, and (iv) determine the frequency of selected healthcare use for foot pain among persons with symptomatic midfoot OA.

## Methods

### Study design

This paper utilises baseline data from a population-based prospective observational cohort study, the Clinical Assessment Study of the Foot (CASF) [12]. Adults aged 50 years and over registered with four general practices in North Staffordshire, United Kingdom, were invited to take part in the study, irrespective of foot-related consultation. Ethical approval was obtained from Coventry Research Ethics Committee (REC reference number: 10/H1210/5) and all participants gave their written consent to participate.

### Data collection

At baseline, eligible participants were mailed a health survey that included general health (Short Form-12 (SF-12)) [13], Hospital Anxiety and Depression Scale (HADS) [14], comorbidities, anthropometric characteristics (self-reported height and weight), foot pain, footwear, healthcare consultation, pain medication use, and demographic and socio-economic characteristics (age, gender, marital status, education, current employment status, and occupation). Foot pain questions included: pain in the foot in the last 12 months; pain, aching or stiffness in the foot in the last month [15], number of days with foot pain in the last 12 months; and the Manchester Foot Pain and Disability Index (MFPDI) [16]. The location of foot pain in the last 4 weeks was ascertained from shading a foot manikin (© The University of Manchester 2000. All rights reserved) [17]. Participants who reported foot pain in the last 12 months and provided written consent to further contact were invited to attend a research assessment clinic where weight-bearing dorso-plantar and lateral radiographs of each foot, clinical interview and physical examination were undertaken, in accordance with defined standardised protocols [12, 18]. Health survey responders were also invited to consent to medical record review.

### Scoring of radiographs and case definitions

Plain radiographs were scored by a single reader (MM) blinded to all other participant information. Osteophytes and joint space narrowing at the 1st and 2nd CMJs, NCJ and TNJ were scored (0–3) according to a validated atlas and classification system [18]. Eight weeks later a random selection of radiographs from 60 participants were re-scored by MM and independently by HBM. Intra-rater and inter-rater reliability for the presence of OA in a midfoot joint were excellent (mean κ = 0.95, mean % agreement = 99 %) and moderate (mean κ = 0.40, mean % agreement = 78 %) respectively.

Midfoot pain was defined as self-reported pain in the last 4 weeks by shading the midfoot region on a foot manikin, designated using a pre-defined regional marking template [17, 19].

Symptomatic midfoot OA was defined as a radiographic score of 2 or more for osteophytes or joint space narrowing on either weight-bearing dorso-plantar or lateral views, in one or more midfoot joints (1st CMJ, 2nd CMJ, NCJ or TNJ), and midfoot pain in the last 4 weeks in the same foot (as defined above).

Disabling symptomatic midfoot OA was defined as symptomatic radiographic OA together with at least one of the ten items within the MFPDI function construct scored at the level of ‘on most/every day(s)’ [20]. If all items were scored at the level of ‘none of the time’ or ‘on some days’, symptomatic midfoot OA was classed as non-disabling. Individuals were defined as having any of the above case definitions if either or both feet were affected.

Individuals identified as having non-specific inflammatory arthritis, rheumatoid arthritis, or psoriatic arthritis were excluded from the analyses based on medical record review (primary care and local hospital) or clinical X-ray report by a consultant musculoskeletal radiologist [5].

### Statistical analysis

#### Estimating population prevalence

Using baseline health survey and radiographic data, the population prevalence of midfoot pain, symptomatic midfoot OA and disabling symptomatic midfoot OA in the individual were estimated using multiple imputation and weighted logistic regression modelling. Multiple imputation was used to account for missing item-level data from the health survey and estimates were then weighted to take into account selective non-response to the health survey [21]. Missing data were inspected to ensure that the missing at random assumption was reasonable.

Imputation involved all baseline responders and utilised the following variables: age, gender, general practice, social class, marital status, number of days with foot pain in the last 12 months, Rasch-transformed MFPDI interval level scores for the pain and function constructs [22], individual MFPDI function items to estimate disabling symptoms [20], self-reported foot pain, aching, or stiffness in the last month, SF-12 score, HADS score, and radiographic foot OA and pain regions. Fifteen imputed datasets were generated and combined using Rubin’s combination rules [23]. Prevalence estimates (and 95 % confidence intervals (CI)) were calculated using the *mim: proportion* command and applied to the total baseline responder population. Selective non-response to the health survey was handled by generating weighted estimates that likely reflect the profile of non-responders using information available for the whole eligible baseline population (age, gender and general practice). Weighted logistic regression was combined with the imputed datasets to generate prevalence estimates (and 95 % CI) in the whole eligible baseline population. Crude population prevalence estimates for midfoot pain, symptomatic radiographic midfoot OA and disabling radiographic symptomatic midfoot OA were then stratified by gender, age, and socio-economic class, based on occupation. This approach to estimating population prevalence mirrors our procedures adopted for estimating overall symptomatic radiographic foot OA [5].

The analyses described below were conducted using data from the CASF clinic cohort. A complete case approach was taken due to the generally very low levels of missing data within clinic participants.

#### Potential aetiological determinants of interest

Binary logistic regression estimated the crude, and as appropriate, age-gender-body mass index (BMI)-adjusted associations between symptomatic midfoot OA and selected variables. Obesity (≥30 kg/m^2^) at time of baseline assessment was calculated from clinic-measured height and weight. Lifetime recall of previous foot and ankle injury was ascertained on standardised personal interview (yes/no response to the question ‘Have you ever injured your feet or ankles?’, with follow-up questions to ascertain type, anatomical location and duration of time since injury, analysed in right feet only). Lifetime recall of frequent use of high-heeled footwear among females was ascertained from a health survey item on footwear [12] (high frequency use was defined as reported use of high-heeled shoes on most days for at least one 10-year period between the age of 20 and 49 years). Nodal interphalangeal joint (IPJ) OA was defined as a Kellgren and Lawrence [24] score of two or more, in two or more IPJs (digits 2–5) and the presence of two or more Heberden or Bouchard nodes (digits 2–5) across either hand [25].

#### Associated impairment and comorbidities

The following self-reported impairment and comorbidities were ascertained from the health survey and their associations with symptomatic midfoot OA examined using binary logistic regression: general health and function (SF-12 physical and mental components, with each variable dichotomised around the median of the data distribution), HADS score (categorised as normal, mild, moderate or severe), chest problems, heart problems, deafness, eyesight problems, hypertension, diabetes, stroke, cancer, circulation problems in the legs, intermittent claudication (defined by the Edinburgh Claudication Questionnaire [26]) and co-occurring joint pain in the last month at other weight-loaded sites (low back, hip, knee, hindfoot/ankle and forefoot). Pain location was defined using recognised body and foot manikins and templates (low back [27]; hip [28]; knee [29]; foot and ankle [17]) that have demonstrated excellent inter- and intra-rater reliability [19, 29]. Crude odds ratios (OR) with 95 % CI were reported together with estimates adjusted for age and gender.

#### Frequency of primary healthcare use

Among adults with symptomatic midfoot OA, the frequency of foot pain-related consultation with a general practitioner (GP) or allied health professional (physiotherapist or podiatrist/chiropodist) was summarised as the 12-month period prevalence, and the proportion of consultations that were with the National Health Service (NHS) or private practice was described. The frequency of medication use for foot pain among adults with symptomatic midfoot OA was summarised as 1-month period prevalence.

All analyses were conducted using STATA V.12.0 (StataCorp, College Station, TX, USA).

## Results

The description of participants recruited into the study has been previously reported [5]. Briefly, in 2010/2011, 9403 potentially eligible adults were identified. Following initial screening, 9194 were posted a health survey, with 5109 responding (adjusted response 56 %). Following further screening, 1634 eligible participants were invited to a research clinic, 560 (34 %) of whom attended. In total, 525 contributed to the final analyses following the exclusion of those with incomplete foot pain data (n = 8), incomplete radiographic data (n = 3) and inflammatory arthritis (n = 24).

### Population prevalence

**Fig. 1:**
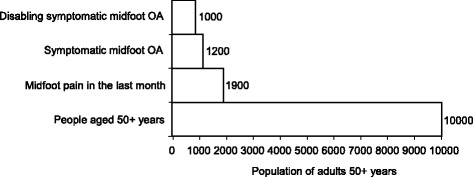
Prevalence staircase for symptomatic midfoot OA

The population prevalence of midfoot pain in the last month among adults aged 50 years and over was 19.4 % (95 % CI: 18.3 %, 20.5 %). The corresponding estimate for symptomatic midfoot OA was 12.0 % (95 % CI: 10.9 %, 13.2 %) and for disabling symptomatic midfoot OA was 9.6 % (95 % CI: 8.6 %, 10.6 %). Based on the imputed estimates, a prevalence staircase was constructed for a denominator population of 10, 000 persons aged 50 years and over (Fig. 1). Prevalence was higher in females, increased most notably in females aged 75 years and over, and was inversely related to socio-economic class (Table 1).

**Table 1:** Population prevalence of midfoot pain, symptomatic, and disabling symptomatic midfoot OA by demographic characteristics

	Midfoot pain	Symptomatic midfoot OA	Disabling symptomatic midfoot OA
All adults aged 50+	19.4 (18.3, 20.5)	12.0 (10.9, 13.2)	9.6 (8.6, 10.6)
Gender			
Male	17.2 (15.7, 18.7)	10.3 (8.9, 11.7)	7.9 (6.7, 9.1)
Female	21.5 (19.9, 23.1)	13.7 (12.0, 15.5)	11.2 (9.7, 12.8)
Age (years) overall			
50–64	20.6 (19.0, 22.2)	11.8 (10.0, 13.5)	9.0 (7.5, 10.4)
65–74	17.6 (15.7, 19.5)	11.1 (9.4, 12.8)	8.8 (7.2, 10.4)
75+	18.7 (16.3, 21.1)	14.4 (11.9, 16.9)	13.0 (10.7, 15.3)
Males			
50–64	18.8 (16.6, 21.0)	10.5 (8.4, 12.6)	7.5 (5.9, 9.1)
65–74	15.1 (12.5, 17.6)	9.3 (6.9, 11.7)	7.8 (5.5, 10.0)
75+	15.4 (11.8, 18.9)	11.3 (8.0, 14.6)	9.9 (6.8, 12.3)
Females			
50–64	22.4 (20.1, 24.7)	13.1 (10.6, 15.5)	10.5 (8.3, 12.6)
65–74	20.2 (17.3, 23.1)	12.9 (10.1, 15.8)	9.9 (7.4, 12.4)
75+	21.1 (17.8, 24.4)	16.6 (13.2, 20.0)	15.3 (12.1, 18.5)
Socio-economic classification			
Managerial and professional	12.7 (10.6, 14.7)	6.9 (4.9, 8.9)	4.9 (3.3, 6.5)
Intermediate occupations	18.8 (16.2, 21.4)	12.6 (9.9, 15.4)	10.5 (8.2, 12.8)
Routine and manual	21.6 (20.0, 23.2)	13.3 (11.6, 15.0)	10.5 (9.1, 11.9)

Based on imputed and weighted analyses

*OA* osteoarthritis

Among clinic attenders, 149 individual feet were diagnosed with symptomatic midfoot OA. Of these, most feet (n = 100, 67 %) had only one joint involved, with the 2nd CMJ being most commonly affected (n = 39, 39 %), followed by the TNJ (n = 36, 36 %), 1st CMJ (n = 13, 13 %) then NCJ (n = 12, 12 %).

### Potential aetiological determinants of interest

Symptomatic midfoot OA was positively associated with BMI. However, for self-reported previous foot or ankle injury, previous frequent use of high-heeled footwear among females and nodal IPJ OA, the 95 % CI for these estimates crossed unity (Table 2). Further analyses of previous foot or ankle injury among right feet suggested that those with symptomatic midfoot OA were more likely to recall having a fracture (Table 3). Examining all foot and ankle injuries to the right foot (n = 42, with 49 injuries), the ankle was reported as the most commonly affected location, particularly for a sprain (41 %), and this was followed by forefoot fracture (16 %) and other forefoot injuries (14 %). Additionally, most recalled injuries occurred over 10 years prior to baseline clinic assessment (56 %) (Table S1 in Additional file 1).

**Table 2:** Relationship between selected potential aetiological determinants of interest and symptomatic midfoot OA

	Total N	SMOA N	Crude OR (95 % CI)	Adjusted OR (95 % CI)
Body mass index (kg/m^2^)				
Non-obese <30	286	49	1	1
Obese ≥30	235	69	2.01 (1.33, 3.05)	2.02 (1.32, 3.08)^a^
Self-reported previous injury to either foot or ankle				
No	166	29	1	1
Yes	359	90	1.58 (0.99, 2.52)	1.60 (0.98, 2.60)^b^
Self-reported frequency of use of high-heeled footwear^c^				
Low	78	18	1	1
High	211	47	0.96 (0.51, 1.77)	0.98 (0.51, 1.88)^d^
Nodal IPJ OA^e^				
No	402	85	1	1
Yes	123	34	1.42 (0.90, 2.26)	1.32 (0.80, 2.16)^b^

Based on complete case clinic data

*OA* osteoarthritis, *SMOA* symptomatic midfoot osteoarthritis, *OR* odds ratio, *CI* confidence interval, *IPJ* interphalangeal joint

^a^Estimate adjusted for age and gender

^b^Estimate adjusted for age, gender and body mass index

^c^Examination was restricted to females and the exposure was defined as previous footwear (low- versus high-heeled shoes) worn on most days for at least one 10-year period between 20 and 49 years old

^d^Estimate adjusted for age and body mass index

^e^Nodal interphalangeal joint (IPJ) OA defined as Kellgren and Lawrence ≥2 in ≥2 IPJs (digits 2–5), and the presence of ≥2 Heberden or Bouchard nodes (digits 2–5) across either hand [25]

**Table 3:** Associations between right symptomatic midfoot OA and previous right foot or ankle injury

Self-reported right foot or ankle injury	Total N	RSMOA N	Crude OR (95 % CI)	Adjusted OR (95 % CI)^a^
One or more previous injury				
No	269	37	1	1
Yes	256	42	1.23 (0.76, 1.99)	1.30 (0.79, 2.12)
One or more previous sprain				
No	342	56	1	1
Yes	167	21	0.73 (0.43, 1.26)	0.80 (0.46, 1.39)
One or more previous fracture				
No	433	59	1	1
Yes	83	20	2.01 (1.13, 3.57)	2.06 (1.14, 3.71)
One or more previous other injury				
No	443	65	1	1
Yes	72	12	1.16 (0.59, 2.28)	1.08 (0.54, 2.15)

Based on complete case clinic data

*OA* osteoarthritis, *RSMOA* right symptomatic midfoot osteoarthritis, *OR* odds ratio, *CI* confidence interval

^a^Estimates adjusted for age, gender and body mass index

### Associated impairment and comorbidities

Strong positive associations were observed between symptomatic midfoot OA and impaired physical function measured by the SF-12, mild and moderate anxiety and mild depression measured using the HADS scores, and diabetes. Following further adjustment for BMI, the positive association with diabetes was attenuated (OR, 1.48; 95 %CI: 0.85, 2.55). Observed associations between symptomatic midfoot OA and four or more comorbidities, and self-reported pain at all weight-loaded joint sites of the body were positive. For co-occurring joint pain, increased magnitude but reduced precision was observed for sites closest to the midfoot (hindfoot/ankle and forefoot) (Table 4). The observed positive associations across all the weight-loaded joint sites remained following further adjustment for BMI (data not shown).

**Table 4:** Associated self-reported impairment and comorbidities among adults with symptomatic midfoot OA

Self-reported impairment/comorbidity	Total N	SMOA N	Crude OR (95 % CI)	Adjusted OR^a^ (95 % CI)
SF-12 Physical impairment^b^				
No	242	30	1	1
Yes	245	76	3.18 (1.99, 5.08)	2.87 (1.78, 4.62)
SF-12 Mental impairment^b^				
No	240	45	1	1
Yes	247	61	1.42 (0.92, 2.19)	1.42 (0.91, 2.21)
HADS anxiety				
Normal (0–7)	289	52	1	1
Mild (8–10)	114	30	1.63 (0.97, 2.72)	1.73 (1.02, 2.94)
Moderate (11–14)	92	29	2.10 (1.23, 3.57)	2.26 (1.30, 3.93)
Severe (15–21)	23	6	1.61 (0.60, 4.28)	1.99 (0.74, 5.39)
HADS depression				
Normal (0–7)	371	71	1	1
Mild (8–10)	86	27	1.93 (1.15, 3.26)	1.86 (1.09, 3.17)
Moderate (11–14)	50	16	1.99 (1.04, 3.80)	1.93 (0.99, 3.75)
Severe (15–21)	11	3	1.58 (0.41, 6.12)	2.11 (0.54, 8.28)
Chest problems				
No	411	87	1	1
Yes	114	32	1.45 (0.91, 2.33)	1.34 (0.83, 2.18)
Heart problems				
No	424	88	1	1
Yes	101	31	1.69 (1.04, 2.74)	1.48 (0.90, 2.43)
Deafness				
No	401	89	1	1
Yes	124	30	1.12 (0.70, 1.80)	0.90 (0.55, 1.49)
Eyesight problems^c^				
No	381	82	1	1
Yes	144	37	1.26 (0.81, 1.97)	1.13 (0.71, 1.78)
Hypertension				
No	284	53	1	1
Yes	241	66	1.64 (1.09, 2.48)	1.40, (0.92, 2.15)
Diabetes				
No	444	90	1	1
Yes	81	29	2.19 (1.32, 3.65)	1.93 (1.14, 3.25)
Stroke				
No	492	113	1	1
Yes	33	6	0.75 (0.30, 1.85)	0.59 (0.23, 1.49)
Cancer				
No	493	109	1	1
Yes	32	10	1.60 (0.74, 3.48)	1.19 (0.54, 2.66)
Circulation problems in legs				
No	358	68	1	1
Yes	167	51	1.88 (1.23, 2.86)	1.52 (0.97, 2.38)
Intermittent claudication^d^				
No	443	94	1	1
Yes	20	4	0.93 (0.30, 2.84)	0.82 (0.26, 2.56)
Non-musculoskeletal comorbidities^e^				
0–1	167	23	1	1
2–3	153	30	1.53 (0.84, 2.77)	1.31 (0.71, 2.41)
4+	137	43	2.86 (1.62, 5.06)	2.23 (1.23, 4.05)
Low back pain				
No	289	49	1	1
Yes	236	70	2.07 (1.36, 3.13)	2.09 (1.37, 3.19)
Hip pain				
No	321	49	1	1
Yes	204	70	2.90 (1.91, 4.41)	3.08 (2.00, 4.74)
Knee pain				
No	293	51	1	1
Yes	232	68	1.97 (1.30, 2.98)	2.12 (1.38, 3.25)
Hindfoot/ankle pain				
No	234	27	1	1
Yes	291	92	3.54 (2.21, 5.68)	3.63 (2.25, 5.86)
Forefoot pain				
No	187	18	1	1
Yes	338	101	4.00 (2.33, 6.86)	4.50 (2.59, 7.82)
Other lower limb pain^f^				
No	77	3	1	1
Yes	448	116	8.62 (2.67, 27.87)	8.53 (2.63, 27.71)

Based on complete case clinic data

*OA* osteoarthritis, *SMOA* symptomatic midfoot osteoarthritis, *OR* odds ratio, *CI* confidence interval, *SF-12* Short Form-12 [13], *HADS* Hospital Anxiety and Depression Scale [14]

^a^Estimates adjusted for age and gender

^b^Variable dichotomised around the median of the data distribution

^c^Excludes the need for glasses

^d^Defined as calf pain when walking at an ordinary pace on level ground (including uphill or when hurried) that disappears in 10 min or less by standing still [26]

^e^Count based on each of the above self-reported variables, excluding SF-12 scores (HADS scores ≥8)

^f^Variable includes presence of pain in hip, knee, hindfoot/ankle or forefoot

### Frequency of primary healthcare use

Most participants with symptomatic midfoot OA consulted a healthcare professional in the last 12 months for foot pain, with the frequency of GP and podiatrist/chiropodist consultations being similar (Table 5). Among individuals with symptomatic midfoot OA in either foot (n = 119), 16 % had accessed private allied health professional healthcare for foot pain in the last 12 months (22 %, n = 5 of physiotherapy consultations, and 25 %, n = 14 of podiatry/chiropody consultations).

**Table 5:** Frequency of selected healthcare use for foot pain

Healthcare use for foot pain	Adults with symptomatic midfoot OA (n = 119)	
	N	Proportion
		% (95 % CI)
Healthcare professional consulted in last 12 months		
GP	55	46.2 (37.1, 55.3)
Physiotherapist	22	18.5 (11.4, 25.6)
Podiatrist/chiropodist	57	47.9 (39.0, 57.0)
Any of the above	86	72.3 (64.1, 80.4)
Pain medication in last month		
Paracetamol	43	36.1 (27.4, 44.9)
Mild/moderate opioids	38	31.9 (23.4, 40.4)
Topical cream/gel/spray	37	31.1 (22.7, 39.5)
NSAIDs, including coxibs	33	27.7 (19.6, 35.9)
Herbal/nutraceuticals	25	21.0 (13.6, 28.4)
Any of the above	84	70.6 (62.3, 78.9)

Based on complete case clinic data

*OA* osteoarthritis, *CI* confidence interval, *GP* general practitioner, *NSAIDs* non-steroidal anti-inflammatory drugs

One-month period prevalence of pain medication use for foot pain was 70.6 % (95 % CI: 62.3, 78.9) (Table 5). One-month period prevalence for one or more form of oral pain medication use (paracetamol, non-steroidal anti-inflammatory drugs, including coxibs and mild/moderate opioids) was 64.7 % (95 % CI: 55.9, 73.4). Pain medication and topical applications were more frequently used than herbal/nutraceutical preparations.

## Discussion

This is the first comprehensive account describing the epidemiology of symptomatic midfoot OA. Our findings suggest that symptomatic midfoot OA is common, being present in an estimated 12 % of the population aged 50 years and over. The higher occurrence of symptomatic midfoot OA in females, older age, and lower socio-economic classes is consistent with previous epidemiological studies of foot pain and other sites of musculoskeletal pain [4, 30].

The associations with obesity, previous injury and pain at all other weight-loaded joint sites but not nodal IPJ OA (a proxy for more widespread OA [31]), together with the load distribution function of the midfoot [9], are consistent with the role of mechanical factors in its pathogenesis. Whilst no one underlying mechanism (e.g. inflammatory or mechanical) appears to be responsible in its entirety for the development of OA [32, 33], the characteristics of deterioration associated with structural and pathological changes suggest that altered biomechanics and joint loading are important modifiable mediators of onset and progression, particularly in the lower limb [34]. At the knee, epidemiological studies have shown malalignment and aberrant loading to be associated with OA development (for example, [35, 36]). As a functional unit, the midfoot is highly sensitive to aberrant biomechanics and altered joint loading due to its load distribution function when walking [37, 38], and our epidemiological findings provide additional evidence in support of a malalignment loading hypothesis. Within the midfoot joints imaged and scored, isolated midfoot joint involvement was the predominant observation, with the 2nd CMJ being the most frequently affected joint, followed by the TNJ. The mechanical vulnerability of the 2nd CMJ as the more rigid apex of the transverse arch [39] appears consistent with a greater susceptibility to aberrant loading compared with the TNJ, which structurally has more functional capacity to accommodate larger gravitational loading effects, together with shear forces during both static and dynamic functional tasks. Different patterns of single joint involvement may reflect different midfoot loading patterns. Although the observed positive associations between symptomatic midfoot OA and obesity extend earlier work, showing that being obese is associated with non-specific foot pain [40], further research is required to confirm or refute a more systemic counterargument for the involvement of obesity [41].

The observed trend that previous injury is associated with symptomatic midfoot OA supports expert clinical opinion that OA changes in the midfoot commonly follow trauma [42] and mirrors a well-established association between injury and OA in the knee [43]. However, more research is needed to better understand the relationships between symptomatic midfoot OA and transient minor traumas versus chronic injury mechanisms and consequences. Whilst footwear selection for females may also precipitate a range of foot-related problems secondary to altered biomechanics [44], the consequences of these alterations may be more pronounced in other regions of the foot, such as the forefoot and toes.

The proportion of participants with symptomatic midfoot OA who reported consulting a GP in the last 12-months for foot pain (46 %) was high compared to previous population estimates generated within North Staffordshire for other joint pain and problems among adults aged 50 years and over (self-reported consultation for knee pain, 33 % in the last 12 months [45]; and self-reported hand problems, 22 % in last 12 months [46]). A record-based review of consultation for musculoskeletal foot problems in the last 18 months among adults aged 50 years and over was previously estimated at 12 % [47]. The higher estimate obtained for consultation for symptomatic midfoot OA seen in our study could possibly arise as a result of concurrent pain elsewhere in the foot contributing to consultation, inaccurate recall of the last 12 months, and consultation rates being higher in a subgroup of self-selecting volunteers who attended a foot research clinic and may have more severe problems.

Nearly three-quarters of participants had recently used some form of pain medication for foot pain. Whilst the frequent use of oral and topical medications would appear in keeping with clinical guidelines for OA in general, one-fifth of participants had recently used herbal or nutraceutical preparations, which is inconsistent with current recommendations [48].

Frequent healthcare use and associated disability must be interpreted at the level of the foot given the high proportion of people with symptomatic midfoot OA who have hindfoot/ankle pain and forefoot pain. However, this study indicates that people with symptomatic midfoot OA may require management and are frequently present in primary care, though often in the context of multimorbidity and not necessarily as a discrete phenotype. The overwhelming majority (97 %) of individuals with symptomatic midfoot OA will report pain at other lower extremity joints and the extent of this comorbidity is greater than expected by chance alone. Shared aetiological pathways would carry implications for clinical care, for example, in addressing common causes of lower extremity pain, in biomechanically based interventions aimed at the lower extremity as a functional unit, and in ‘collateral benefits’ to lower extremity pain of effective local treatment to one joint. For the local management of symptomatic midfoot OA, there is limited evidence for interventions such as foot orthoses [49], intra-articular corticosteroid/local anaesthetic injections [50] and arthrodesis [51]. Better characterisation of this phenotype may help to inform more effective targeted treatments.

Strengths of this study include census sampling from general practice registers and the use of multiple imputation and weighted logistic regression to account for selection bias. Although our approach adopts recognised statistical adjustment techniques [21], these estimates should be viewed cautiously due to non-response. Whilst causality cannot be inferred for obesity, OA at the knee has been shown to cumulate through adulthood with increased exposure to elevated BMI [52], and reverse association (symptomatic midfoot OA causing weight gain) seems less plausible. The majority of self-reported injuries occurred over 10 years ago and high-heeled footwear exposure (between 20 and 49 years old) predated the sample age (≥50 years old), suggesting that these exposures appear to predominantly occur earlier in life.

The study has some noteworthy limitations. The clinical sample all had foot pain in the last 12 months. Therefore, prevalence estimates represent symptomatic individuals and all associations are relative to foot pain elsewhere. Consequently, observed associations may underestimate the true effect. Despite excellent intra-rater reliability for scoring the presence of OA in a joint, inter-rater reliability was moderate. Furthermore, we noted that the main scorer MM was systematically more conservative compared to HBM. Therefore, the reported population prevalence estimates may be underestimated. Also, estimates for previous injury, previous footwear, comorbidity and healthcare use were derived from self-report data, and laterality for co-occurring joint pain was not considered. The footwear question designed specifically for this study has not been validated. Both footwear and previous injury may be particularly vulnerable to recall bias, however, attempts were made to minimise these issues. First, in the standardised clinical interview all participants were probed in relation to a variety of previous injury exposures over a number of questions to promote and assist recall. Second, previous footwear was asked of all participants in the health survey irrespective of foot pain status. Finally, the selected healthcare use estimates are based on small numbers and therefore could only be estimated imprecisely.

## Conclusions

Symptomatic midfoot OA occurs commonly in the population aged 50 years and over. Its relationship with demographic and socio-economic factors is similar to other forms of OA but the pattern of association with potential determinants, together with the form and function of the midfoot, suggest a central role for mechanical load in this phenotype. As such it may provide a useful model for further studies of mechanical load in OA pathogenesis. Clinical research could focus on the effectiveness of existing and emerging non-pharmacological treatments for this phenotype.

## Additional file

Additional file 1: Table S1. Foot or ankle injury location and duration of time since injury among adults with symptomatic midfoot OA in the right foot.

## Abbreviations

BMI: body mass index
CASF: Clinical Assessment Study of the Foot
CI: confidence interval
CMJ: cuneometatarsal joint
GP: general practitioner
HADS: Hospital Anxiety and Depression Scale
IPJ: interphalangeal joint
MFPDI: Manchester Foot Pain and Disability Index
MTPJ: metatarsophalangeal joint
NCJ: navicular first cuneiform joint
NHS: National Health Service
NSAIDs: Non-steroidal anti-inflammatory drugs
OA: osteoarthritis
OR: odds ratio
SF-12: Short Form-12
TNJ: talonavicular joint
