# Supplementary material for: The epidemiology of symptomatic midfoot osteoarthritis in community-dwelling older adults: cross-sectional findings from the Clinical Assessment Study of the Foot
Source: Arthritis Res Ther. 2015 Jul 13;17(1):178. doi: 10.1186/s13075-015-0693-3 (PMC4499901; doi:10.1186/s13075-015-0693-3)
Supplement: Additional file 1: Table S1. — Foot or ankle injury location and duration of time since injury among adults with symptomatic midfoot OA in the right foot. [file 13075_2015_693_MOESM1_ESM.docx]

**Additional file 1: Table S1.** Foot or ankle injury location and

duration of time since injury among adults with

symptomatic midfoot OA in the right foot.

| **Total injuries by type** | **RSMOA**  **(n=42 persons)** | |
| --- | --- | --- |
|  | **Location** | **N (%)** |
| Sprain | Ankle  Midfoot  Forefoot | 20 (41)  1 (2)  0 (0) |
| Fracture | Ankle  Heel  Midfoot  Forefoot | 5 (10)  2 (4)  3 (6)  8 (16) |
| Other injury^a^ | Ankle  Heel  Midfoot  Forefoot | 3 (6)  0 (0)  0 (0)  7 (14) |
| Total injuries |  | 49 |

|  | **When?** | **N (%)** |
| --- | --- | --- |

| Sprain | <1 yr  1 to <5 yrs  5 to <10 yrs  10+ yrs | 1 (2)  5 (9)  2 (4)  14 (26) |
| --- | --- | --- |
| Fracture | <1 yr  1 to <5 yrs  5 to <10 yrs  10+ yrs | 1 (2)  5 (9)  5 (9)  9 (17) |
| Other injury^a^ | <1 yr  1 to <5 yrs  5 to <10 yrs  10+ yrs | 2 (4)  0 (0)  3 (6)  7 (13) |
| Total injuries |  | 54 |

Based on complete case clinic data

RSMOA, right symptomatic midfoot osteoarthritis

^a^Includes trauma such as crush injuries, impact injuries and

lacerations
